# Secretions from seminal vesicles lack characteristic markers for prostasomes

**DOI:** 10.3109/03009730903366067

**Published:** 2010-04-07

**Authors:** Göran Sahlén, Ove Nilsson, Anders Larsson, Lena Carlsson, Bo Johan Norlén, Gunnar Ronquist

**Affiliations:** ^1^Department of Urology, University Hospital, UppsalaSweden; ^2^Department of Medical Cell Biology, Biomedical Center, UppsalaSweden; ^3^Department of Clinical Chemistry, University Hospital, UppsalaSweden

**Keywords:** CD10, CD13, CD26, clusterin, electron microscopy, HSP70, markers, prostasomes, seminal-vesicle

## Abstract

**Background:**

Prostasomes are suggested to be produced in the prostate gland. Although biochemical studies support this, some immunohistochemical findings indicate that also the seminal vesicles could be a source of prostasomes. Therefore, we have compared the secretion of the vesicles with that of the prostate using biochemical and ultrastructural techniques.

**Methods:**

Ultracentrifuged pellets of substance from seminal vesicle secretions were analysed by sodium dodecyl sulfate-polyacrylamide gel electrophoresis (SDS-PAGE) and flow cytometry. The secretory cells of the seminal vesicles were examined with transmission electron microscopy. These findings were then compared with published results from similar studies of the prostate secretory cells.

**Results:**

In SDS-PAGE, the seminal vesicle pellets lacked the three prostasome-characteristic CD-markers, namely CD10, CD13, and CD26, but expressed two proteins of about 55 kDa and 70 kDa, corresponding to clusterin and heat shock protein (HSP70). Flow cytometry showed the presence of secretion particles in the seminal pellet, although of a smaller size than that of the prostasomes. Electron microscopy of the luminal part of the cells in the seminal vesicles demonstrated many secretion granules, each enclosed in a vesicle with a size of about 1 μm.

**Conclusions:**

Pelleted seminal vesicle secretion is different to prostate secretion in several ways. No prostasome characteristics were detected in the pelleted seminal vesicle secretion.

## Introduction

The major accessory reproductive glands in man are the seminal vesicles and the prostate gland. In adulthood, androgens stimulate a functional differentiation to secretory activity of these glands. The seminal vesicles are derivatives of the Wolffian duct and are lined by a pseudostratified epithelium consisting of principal cells with apical secretory vesicles each containing a secretion granule (1–3). The prostate gland is a derivative of the embryonic Mullerian duct and lined by cylindrical cells containing apical secretory vesicles. In such vesicles there are scattered small granules, named prostasomes (4,5). A notable clinical experience is that a primary malignancy in the epididymis or in the seminal vesicles is very rare, although the incidence of malignancy in the prostate gland is notoriously high.

In seminal plasma and prostate secretions, Ronquist et al. (6) found Mg^2+^- and Ca^2+^-dependent ATPase associated with a pellet containing many small granules and vesicles. Brody et al. (7) demonstrated in a detailed examination by electron microscopy that they had a size of 40–500 nm. These components were named prostasomes by the authors. Later, our high-resolution studies (magnification about 35,000×) with electron microscopy showed that the prostasomes demonstrated various shapes and internal structures in the form of mixtures of membraneous, dense, and dark areas (4,8). The prostasome structure was also analysed with conventional and cryo-electron microscopy, confirming the complex appearance of prostasomes (9).

Monoclonal antibodies against prostasomes were raised by Nilsson et al. (10), and this opened for immunohistochemical studies of their distribution. The monoclonal antibodies labelled the apical parts of the secretory cells in the prostate epithelium and the secretion in the prostate gland ducts, but they did not react with blots of prostate-specific antigen (PSA), nor with prostate acid phosphatase (PAP) (11). One monoclonal antibody was analysed and was found to detect dipeptidyl peptidase IV (CD26) (12). This enzyme is a membrane-bound prostasome enzyme present in the prostate secretion but absent in the fluid from the seminal vesicles (13,14). Thus, this monoclonal antibody recognized prostasomes. The antibody, however, showed cross-reactivity with syncytiotrophoblasts in first trimester placenta, exocrine cells of the pancreas, some cells in pancreatic islets, and weakly with epithelial cells in the seminal vesicle.

A subsequent paper reported studies with a polyclonal rabbit-derived antiserum against purified human seminal prostasomes (15). This antibody registered immunoreactivity in the apical region of the secretory cells in the prostate epithelium and in the luminal secretion. However, a reaction was reported also in the epididymis and the seminal vesicles. Further, the antibody showed reactions in the bile canaliculi, the human liver, the collecting ducts in the kidney, the parietal cells of the stomach, and the acinar cells of the parotid and submandibular glands. The authors concluded that these findings suggested that prostasomes were not necessarily a unique secretory product of the prostate gland, but could derive also from the seminal vesicles or the epididymis. So, the issue of the origin of the prostasomes is still on the table.

The purpose of this paper is to find out whether the differences between the seminal vesicles and the prostate in terms of biochemistry and ultrastructure of their secretory products could settle the question of the origin of the prostasomes.

## Materials and methods

### Patients

With the approval of the Ethics Committee of the University Hospital in Uppsala, and after informed consent by the patients, ten men were included in this study. They were included consecutively as patients scheduled for major open surgery which would expose the seminal vesicles. The patients underwent radical retropubic prostatectomy because of prostate cancer. Preoperatively they were all regarded as having localized disease.

Seven patients had no palpable tumours (T1c), and three patients had palpable ones although restricted to the prostate (T2). In the postoperative histology reports, two cases of extra-capsular growth were found, but in none of the ten cases was there any tumour growth of prostate cancer into the seminal vesicles.

### Preparation of seminal vesicle secretion

The seminal vesicles were exposed in the operating field, and before the vascular supplies to the vesicles were severed, secretion fluid was aspirated with a large-bore syringe. The secretion was dissolved in cold 30 mmol/L Tris-HCl buffer at pH 7.6 made isotonic with sodium chloride for subsequent biochemical examination. The vesicle fluids were pooled and processed by differential centrifugation and preparative ultracentrifugation according to the procedures for prostasomes (16). The pellet was saved and frozen at -70°C for later analyses. The results were then compared with known corresponding characteristics of prostasome pellets from seminal plasma.

### SDS-PAGE and immunoblotting

SDS-polyacrylamide gel electrophoresis (SDS-PAGE) and immunoblotting were performed with Bio-Rad systems (Hercules, CA, USA) according to the instructions of the manufacturer. Pelleted substance from seminal vesicle secretion was loaded on a 12%–14% polyacrylamide gel. The proteins were visualized by Coomassie staining (20% ethanol, 5% acetic acid, and 0.07% Coomassie Brilliant Blue R 250; Bio-Rad).

### Flow cytometry

Pooled samples were analysed utilizing an Epics Profile XL-MCL cytometer (Coulter Electronics, Hialeah, FL, USA). A total of 100 μL of sample were analysed each time for determination of particle concentration and size. For analyses of CD-markers as well as of heat shock protein (HSP)-70 and clusterin, data processing from 5000 particles was carried out with the XL software (Coulter Electronics). Based on light-scattering properties, each particle was represented by a point in a rectangular co-ordinate system. The location and size of the gate was set in accordance with highly purified prostasomes (16). Discrimination frames were placed around the particle cluster using forward and side scatter. The instrument gave percentage of positively stained particles, median and mean fluorescence intensity (MFI), and complexity (right angle scatter). Analytical markers were set in the fluorescence channel to measure MFI. The gates were set prior to the examination. The experiments were run five times.

### Preparations for electron microscopy

Biopsies of the seminal mucosa were taken during radical prostatectomy. The biopsies were quickly put in a fixative of 2.5% glutaraldehyde in phosphate-buffered saline (pH 7.2). After fixation, the specimens were embedded in Epon to form plastic blocks according to conventional techniques. The plastic blocks were cut in 2 μm sections for light microscopy to find suitable areas. These areas were trimmed to be cut in 50 nm sections for ultrastructural examinations. The sections were put on slot grids with 0.5% Formvar film, contrasted with lead citrate and uranyl acetate, and examined in an electron microscope (Hitachi H-7100).

## Results

### SDS-PAGE

**Figure 1.**
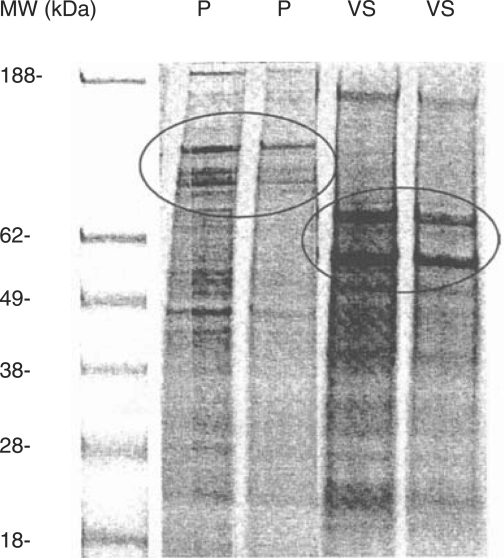
SDS-polyacrylamide electrophoresis (SDS-PAGE) of seminal prostasomes (P) and vesicular seminalis secretion (VS) at two different concentrations. Molecular markers in lane 1 (MW). Prostasome marking CD proteins (at 150, 120 and 90 kDa) encircled in the SDS-PAGE pattern lanes marked P. HSP70 and clusterin (at 70 and 55 kDa) encircled in SDS-PAGE pattern lanes marked VS.

Colloidal-blue stained SDS-PAGE gels of separated proteins were obtained at two different concentrations of both purified prostasomes and pellets from seminal vesicle secretion. The typical banding pattern for prostasomes in the high molecular weight range was recognized with three distinct bands at 150, 120, and 90 kDa (Figure 1). These bands were identified as aminopeptidase N (CD13), dipeptidyl peptidase IV (CD26), and enkephalinase (CD10) (16), respectively. Pellets from the seminal vesicle secretion were distinguished from prostasomes by lacking these three bands. Instead, two other protein bands were over-expressed, one corresponding to a molecular weight of about 70 kDa and the other to about 55 kDa.

### Flow cytometry

**Figure 2.**
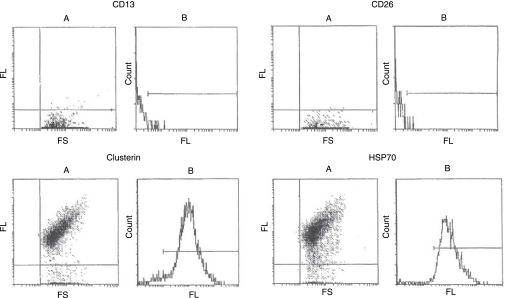
Flow cytometric detection of membrane-bound proteins on particles in the secretion from the seminal vesicle. A: Forward scatter (FS) versus fluorescence (FL). B: Fluorescence (FL) versus number of events (counts). CD13 (aminopeptidase N) and CD26 (dipeptidyl peptidase IV), which are two prostasome-bound antigens, did not give any positive reactions, while CD55 (clusterin/apolipoprotein J) and CD70 (heat shock protein-70) showed distinct reactions.

Flow cytometric analyses of pelleted substance from seminal vesicle secretion revealed particles somewhat smaller than prostasomes. Application of monoclonal antibodies against dipeptidylpeptidase IV (CD26) and aminopeptidase N (CD13) to the flow cytometer did not give rise to any immunoreaction in the preparation from seminal vesicles. This indicated a lack of these typical markers for prostasomes in the seminal vesicles. In the SDS-PAGE, two bands at 70 and 55 kDa were over-expressed in seminal vesicle secretion as opposed to purified prostasomes. To evaluate this finding, monoclonal antibodies against a heat shock protein, HSP70, and clusterin/apolipoprotein J were applied resulting in distinct reactions to both proteins (Figure 2).

### Ultrastructure of secretory components in the seminal vesicle epithelium

The seminal vesicle epithelium contained columnar secretory cells with apical portions bulging into the lumen of the gland ducts. The apical parts of the cells were dominated by vesicles, each containing a single, dense secretion granule with a size of about 0.3 μm. The granules did not quite fill the vesicle. Some granules showed a substructure of small particles. The secretion in the gland ducts appeared as an amorphous substance containing scattered areas with small groups of particles, located among the microvilli of the secretory cells. No structures similar to prostasomes were observed in the seminal vesicles.

## Discussion

The secretory products of the epithelial cells in the vesicles and the prostate differ a great deal. In split ejaculate fractions, which separate the early prostate-derived portion of the ejaculate from the later fraction from the seminal vesicles, the biochemical composition of the different fractions is characteristic of the organ from which they emanate. The seminal vesicles produce a fluid characterized by a high content of fructose, prostaglandins, and semenogelins (17–19). The prostate has a secretion rich in citric acid, zinc, calcium, magnesium, and proteins like prostate-specific antigen (PSA), prostatic acid phosphatase (PAP), beta-microseminoprotein (beta-inhibin), cystatin C, and insuline-like growth factor binding protein 2 (IGFBP-2) (20–24).

A polyvalent rabbit-derived antibody against prostasomes was reported to react strongly with cells in epididymis and with apical parts in the secretory cells of the prostate epithelium. There were no reactions in the testis (15). This was proposed to indicate a source of prostasomal components in the epididymis. However, although the prostasome is a small granule, it has been reported to harbour more than 100 identified proteins as demonstrated by mass spectrometry (9,24). This indicates the complexity of the prostasome proteins and a subsequent increasing possibility of cross-reactions. Thus, findings obtained by immunohistochemistry alone cannot be taken as proof for the site of origin of prostasomes. Further, the enzymatic activity in seminal plasma of a prostasome-bound, Ca^2+^/Mg^2+^-dependent ATPase was not diminished when the ejaculates in 13 men, before and after vasectomy, were examined (25). Thus, this rules out the contribution of prostasomes from the testis and epididymis.

Cluster of differentiation (CD) proteins are individual molecules recognized by a series of monoclonal antibodies. Three such CD proteins (CD10, CD13, and CD26) serve as markers of prostasomes in seminal plasma. Enkephalinase (CD10) is an enzyme restricted to the prostate secretory cells and their secretion, and it binds a polyclonal antibody against prostasomes (26). Aminopeptidase N (CD13) is linked to the prostasome membrane (27), and dipeptidyl peptidase IV (CD26) is recognized by a monoclonal antibody against prostasomes (12). When comparing pellets of prostasomes with the pelleted substance from seminal vesicle secretion, we observed a difference in properties between the two samples. The SDS-PAGE patterns demonstrated that the prostasome-typical banding with CD10, CD13, and CD26 was lacking completely in the pellet of substance from seminal vesicle secretion. In the seminal vesicle material, however, there was a distinct expression of two proteins recognized by monoclonal antibodies against clusterin (CD55) and heat shock protein-70 (CD70). This pattern was not observed in the prostasome pellets. Thus, we have not acquired any indication that prostasomes are present in the vesicle secretion.

The biochemical differences between the secretions of the seminal vesicles and the prostate were accompanied by structural differences found by the present electron microscopical studies. They revealed that the secretion in the seminal vesicle epithelium appeared as single granules, each one being enclosed in a vesicle, while the secretion in the prostate epithelium has been noticed as many, much smaller granules and vesicles (4), that is, prostasomes which were stored in the secretion vesicles. Thus, no secretory structures similar to the prostasomes were observed in the secretory cells of the seminal vesicle.

Flow cytometry disclosed that the secretion from the seminal vesicles contained small particles, smaller than the prostasomes. Since our electron microscopy findings suggest a disintegration of the secretion granules in the vesicle gland lumina, it seems that the secretion granules fall apart in the gland ducts, generating the small particles.

Our high-resolution micrographs of the prostasomes demonstrated that they were membrane-bound with an interior of dark, dense, and membraneous substances in various shapes and proportions (4,8). Similar structures were also noticed at the Golgi apparatus. A recent report on the prostasome ultrastructure, obtained by transmission electron microscopy and cryo-electron microscopy (9), confirmed our findings that the prostasomes display an irregular inner structure. However, it has to be realized that prostasomes, which have passed through procedures for purification and/or preparation for electron microscopy, have lost and changed various components and by this also lost their native properties. Thus, no biochemical or similar conclusions should be based solely on observations by transmission electron microscopy on this type of material.

In conclusion, our findings show that secretions from seminal vesicles lack prostasomes.
